# Interaction of ApoA-IV with NR4A1 and NR1D1 Represses G6Pase and PEPCK Transcription: Nuclear Receptor-Mediated Downregulation of Hepatic Gluconeogenesis in Mice and a Human Hepatocyte Cell Line

**DOI:** 10.1371/journal.pone.0142098

**Published:** 2015-11-10

**Authors:** Xiaoming Li, Min Xu, Fei Wang, Yong Ji, W. Sean DavidsoN, Zongfang Li, Patrick Tso

**Affiliations:** 1 National Local Joint Engineering Research Center of Biodiagnostics and Biotherapy, The Second Affiliated Hospital of Medical College, Xi’an Jiaotong University, 157 W 5th Rd, Xincheng, Xi'an, Shaanxi, 710004, China; 2 Department of Pathology and Laboratory Medicine, Metabolic Diseases Institute, University of Cincinnati, 2180 E. Galbraith Road, Cincinnati, 45237–0507, United States of America; Northeast Ohio Medical University, UNITED STATES

## Abstract

We have previously shown that the nuclear receptor, NR1D1, is a cofactor in ApoA-IV-mediated downregulation of gluconeogenesis. Nuclear receptor, NR4A1, is involved in the transcriptional regulation of various genes involved in inflammation, apoptosis, and glucose metabolism. We investigated whether NR4A1 influences the effect of ApoA-IV on hepatic glucose metabolism. Our *in situ* proximity ligation assays and coimmunoprecipitation experiments indicated that ApoA-IV colocalized with NR4A1 in human liver (HepG2) and kidney (HEK-293) cell lines. The chromatin immunoprecipitation experiments and luciferase reporter assays indicated that the ApoA-IV and NR4A1 colocalized at the RORα response element of the human *G6Pase* promoter, reducing its transcriptional activity. Our RNA interference experiments showed that knocking down the expression of NR4A1 in primary mouse hepatocytes treated with ApoA-IV increased the expression of NR1D1, G6Pase, and PEPCK, and that knocking down NR1D1 expression increased the level of NR4A1. We also found that ApoA-IV induced the expression of endogenous NR4A1 in both cultured primary mouse hepatocytes and in the mouse liver, and decreased glucose production in primary mouse hepatocytes. Our findings showed that ApoA-IV colocalizes with NR4A1, which suppresses *G6Pase* and *PEPCK* gene expression at the transcriptional level, reducing hepatic glucose output and lowering blood glucose. The ApoA-IV-induced increase in NR4A1 expression in hepatocytes mediates further repression of gluconeogenesis. Our findings suggest that NR1D1 and NR4A1 serve similar or complementary functions in the ApoA-IV-mediated regulation of gluconeogenesis.

## Introduction

Metabolic diseases, such as obesity and type 2 diabetes, are a major cause of morbidity and mortality in industrialized nations worldwide. Previous studies have shown that the nuclear receptors (NRs), NR4A1 (also known as Nur77) and NR1D1, contribute to the pathology of metabolic disease through their role in ligand-dependent regulation of cellular glucose metabolism [1]. The NR1D1 and NR4A1 proteins belong to a superfamily of structurally related ligand-dependent transcription factors that have been designated as orphan NRs because no endogenous ligands have been shown to mediate their effects on glucose homeostasis.

The transient expression of NR4A1 is rapidly induced by a diverse range of stimuli, including mechanical stress [2], exercise [3], cAMP activation [4], protein kinase A, protein kinase C [5], G protein-coupled receptor [6], mitogen-activated protein kinase [7], and tyrosine kinase signaling pathways[8]. Members of the NR4A subgroup of NRs are involved in various physiological processes, including cardiovascular disease [9], steroidogenesis [10], inflammation [11], type 2 diabetes [12], and metabolic syndrome [13]. After feeding on a high-fat diet, NR4A1-null mice display major metabolic changes, including greater weight gain, lower energy, increased insulin resistance, and a slower blood glucose clearance rate, compared with healthy mice [14], and these types of molecular and cellular events have been shown to be associated with reduced NR4A1 expression both *in vivo* and *in vitro*.

Synthesized and secreted primarily by enterocytes of the small intestine [15], ApoA-IV is involved in the intestinal absorption of lipids and lipid-soluble vitamins [16], free cholesterol efflux [17], and the inhibition of lipoprotein peroxidation [18]. The ApoA-IV protein is a major component of high-density lipoprotein (HDL) and chylomicrons. During the postprandial state, ApoA-IV in chylomicrons rapidly equilibrates with its lipid-free form in plasma [19]. In our previous study, we showed that elevated ApoA-IV expression increases insulin secretion, lowering blood glucose [20]. The mechanism through which ApoA-IV mediates glucose metabolism in hepatocytes has remained unclear.

Also known as REV-ERBα, NR1D1 is involved in the regulation of various pathways involved in energy homeostasis [21,22]. The binding of heme and NR1D1 represses hepatic gluconeogenesis [22]. In our recent previous study, we found that ApoA-IV inhibits hepatic gluconeogenesis by stimulating the expression of NR1D1 and interacting directly with NR1D1 to downregulate the expression of the key gluconeogenic genes, *PEPCK* and *G6Pase* [23]. ApoA-IV also mediates increases in the serum level of insulin, and inhibits hepatic gluconeogenesis both *in vitro* and *in vivo* [20]. Because NR4A1 has also been shown to function in hepatic glucose homeostasis [14], we investigated whether NR4A1 is involved in the ApoA-IV-mediated regulation of hepatic glucose metabolism. We found that ApoA-IV colocalizes with NR4A1, and that their interaction regulates the transcription of key genes involved in hepatic glucose homeostasis.

## Materials and Methods

### Cell lines and cell culture

The HEK-293 and HepG2 cells were obtained from ATCC (Manassas, VA, USA), and grown in Dulbecco’s Modified Eagle Medium (DMEM) supplemented with 10% fetal bovine serum (FBS) and 1% penicillin/streptomycin in an atmosphere of 5% CO_2_. The cells were passaged 2 to 3 times per week. Only cells in the exponential growth phase were used in our experiments.

### Quantitative reverse transcription and real-time PCR (qRT-PCR)

Total RNA was isolated using the RNeasy Mini Kit (Qiagen, Hilden, Germany). First-strand cDNA was synthesized from 1μg of total RNA using the Scripts cDNA Synthesis Kit (Bio-Rad Laboratories, Hercules, CA, USA), according to the manufacturer’s instructions. Real-time PCR was performed in 25-μL reactions using iQ SYBR Green Supermix (Bio-Rad) and the iCycler iQ Detection System (Bio-Rad), as previously described [23]. All of the primers used for qRT-PCR were obtained from Integrated DNA Technologies (Coralville, IA, USA). The amount of the target mRNA measured was normalized relative to the level of cyclophilin mRNA.

### Western blotting

Western blotting was performed using immunoblotting reagents purchased from Cell Signaling Technology (Beverly, MA, USA), according to the protocol provided by the manufacturer. Relative levels of protein expression were determined by normalizing the results for the target protein to those of GAPDH.

### Immunofluorescence and confocal microscopy

Approximately 1.2 × 10^6^ HepG2 cells were transfected with 4 μg of human *NR4A1* plasmid DNA per electroporation using the Nucleofector Kit V (Amaxa, Gaithersburg, MD, USA), according to the manufacturer’s instructions, and cultured for 30 h. The transfected cells were seeded in 8-well chamber slides. Following a 30-h growth period, the transfected cells were fasted in DMEM with 1% FBS for 16 h. The cells were treated for 2 h with a recombinant fusion protein (20 μg/mL) consisting of human ApoA-IV (r-h-apoA-IV) and green fluorescent protein (GFP), which had been purified as described previously [23]. The cells were fixed in the chamber slides using 4% paraformaldehyde, and permeabilized with 0.2% Triton X-100. Non-specific binding was blocked with 5% normal goat serum (Sigma-Aldrich, St. Louis, MO, USA). The cells were incubated with 1:200 rabbit anti-human NR4A1 (Santa Cruz Biotechnology, Dallas, TX, USA) and 1:200 mouse anti-GFP (Cell Signaling Technology) primary antibodies overnight at 4°C, followed by incubation with 1:200 Alex Flour-594-conjugated goat anti-rabbit (Invitrogen) and 1:200 fluorescein-isothiocyanate (FITC)-conjugated goat anti-mouse secondary antibodies (Invitrogen). The cells were viewed using a Zeiss LSM-510 confocal fluorescence microscope.

### *In situ* proximity ligation assay (PLA)

Approximately 5 × 10^4^ HEK-293 cells were seeded in 8-well chamber slides for 24 h, and the cells were transfected with 150 ng of human *NR4A1* plasmid DNA (Origene, Rockville, MD, USA) using the Lipofectamine 2000 reagent (Invitrogen, Carlsbad, CA, USA), according to the manufacturer’s instructions. HepG2 or HEK-293 cells were transfected with the human *NR4A1* plasmid for 48 h, followed by treatment with r-h apoA-IV-GFP for 2 h. The transfected cells were fixed and permeabilized as described above for the immunofluorescence analysis. Negative control cells were transfected with the NR4A1 plasmid, and treated with GFP. The PLA was performed using the Duolink II PLA probe anti- Mouse PLUS and Duolink II PLA probe anti-Rabbit MINUS, and Duolink II Detection Reagents Red (Sigma-Aldrich, Shanghai, China). The cells were blocked using the Duolink blocking solution. The slides were incubated with 1:200 rabbit anti-human NR4A1 and 1:200 mouse anti-GFP primary antibodies, as described above. Treatment with primary antibody was omitted from one set of slides as an additional negative control. The slides were incubated with the Duolink PLA probes, which consisted of two oligonucleotide-conjugated anti-mouse and anti-rabbit secondary antibodies, at 37°C in a humidified chamber for 2 h. The cells were incubated in hybridization solution, which contained two oligonucleotide linkers that were complementary to the oligonucleotide labels of the PLA probes, at 37°C for 15 min in a humidified chamber. The slides were washed, and incubated at 37°C for 15 min in the Duolink ligation solution, which contained DNA ligase that covalently bonded the hybridization linkers to the PLA probe oligonucleotides, forming a circular DNA molecule that connected the two different secondary antibodies. The slides were incubated for 90 min in the Duolink amplification solution, which contained a polymerase that synthesizes a concatemeric oligonucleotide product of the DNA sequence that linked the secondary antibodies by rolling circle DNA amplification. The slides were washed, and incubated for 60 min in the Duolink detection solution, which contained fluorescently labeled oligonucleotides that hybridized to the rolling circle amplification product. The slides were washed with decreasing concentrations of sodium citrate buffer, followed by washing in 70% ethanol. The cells were mounted with mounting media (Santa Cruz Biotechnology) containing diamino-2-phenyl-indole (DAPI) for nuclear staining. The colocalization of ApoA-IV with NR4A1 was represented by red fluorescence, which was visualized using a Zeiss Axiovert 200 fluorescence microscope.

### Coimmunoprecipitation

To coimmunoprecipitate ApoA-IV and other proteins directly associated with it, HepG2 cells were incubated in DMEM with 1% FBS overnight, and treated with r-h-apoA-IV-GFP or GFP (negative control) for 6 h. The nuclear proteins were extracted from the cells using the NE-PER Nuclear Extraction Reagents (Thermo Fisher Scientific, Waltham, MA, USA), according to the manufacturer’s instructions. An aliquot containing 100 μg of nuclear proteins was combined with 25 μL of protein G magnetic beads and 3 μg of anti-GFP antibody, and incubated at 4°C. The beads were washed, and the immunoprecipitates were eluted using 2× SDS loading buffer. The eluates were heated at 95°C for 10 min, and analyzed by SDS-PAGE and western blotting with anti-NR4A1 and anti-ApoA-IV antibodies (Santa Cruz Biotechnology) or an anti-GFP antibody (control).

### Chromatin immunoprecipitation (ChIP) assay

To determine whether the ApoA-IV-NR4A1 complex bound to regulatory elements of the *G6Pase* promoter, the ChIP assay was performed using the ChIP-IT Express Enzymatic Kit (Active Motif, Carlsbad, CA, USA), according to the manufacturer’s instructions. HepG2 cells were incubated in DMEM with 1% FBS overnight, and treated with r-h-apoA-IV-GFP or GFP (control) for 6 h. Immunoprecipitation was performed at 4°C overnight using anti-NR4A1 and anti-ApoA-IV antibodies. Primers were used to amplify the sequence of the RORα response element (RORE) in the human *G6Pase* promoter or a sequence in the *GAPDH* promoter (control) by PCR, as described previously [23].

### Luciferase activity assay

HEK-293 cells were grown in 24-well plates, and the cells were transfected for 24 h with 0.3 μg of the *G6Pase*-luciferase reporter plasmid, 0.6 μg of the human *NR4A1* plasmid, 5 ng of the *Renilla* luciferase reporter plasmid, and the anti-NR4A1 siRNA (siNR4A1), control siRNA, or an equivalent volume of solvent (vehicle control) using Lipofectamine 2000, according to the manufacturer’s instructions. Cells were also transfected with the *G6Pase*-luciferase and *Renilla* luciferase reporter plasmids and the pcDNA3.1 plasmid as a control. The transfected cells were treated with recombinant human ApoA-IV protein or vehicle control for 24 h. Luciferase activity was measured using the Dual-Luciferase Reporter Assay System (Promega, Madison, WI, USA), and the relative activity was determined by dividing the number of light units generated by firefly luciferase by that of renilla luciferase in the same reaction.

### Analysis of the knockdown of NR1D1 and NR4A1 expression in primary mouse hepatocytes

Primary mouse hepatocytes were isolated from anesthetized 3-month-old male C57BL/6J mice, as described previously [22], and the mice were sacrificed by cervical dislocation. The siNR1D1 (Cat. no. SASI_Mm01_00116940), which targeted the mouse NR1D1 mRNA, and the siNR4A1 (Cat. no. SASI_Mm01_00077215), which targeted the mouse NR4A1 mRNA, were purchased from Sigma-Aldrich. The recombinant mouse ApoA-IV protein (r-m-apoA-IV) was purified, as described previously [23]. Approximately 1.2 × 10^6^ primary mouse hepatocytes were transfected for 48 h with 30 pmol of siNR4A1 or control siRNA per electroporation using the Nucleofector Kit V (Amaxa), according to the manufacturer’s instructions. The cells were fasted in DMEM with 1% FBS for 16 h, followed by treatment with 20 μg/mL r-m-apoA-IV. The level of glucose in the medium was measured at 24 h posttreatment using the glucose assay reagent (Diagnostic Chemicals, Charlottetown, Canada), and normalized relative to the concentration of total cellular protein. The levels of the PEPCK and G6Pase mRNAs were measured by qRT-PCR at 6 h posttreatment. The levels of the NR4A1 and GAPDH (control) proteins were assessed by western blotting at 6 h posttreatment. Primary mouse hepatocytes were transfected with 30 pmol of siNR4A1, siNR1D1, or control siRNA (siC) for 48 h, as described above. The levels of the NR1D1 and NR4A1 mRNAs were measured using qRT-PCR, and the levels of the NR1D1, NR4A1, and GAPDH (control) proteins were measured by western blotting using anti-NR4A1, anti-NR1D1 (Cell Signaling Technology), and anti-GAPDH (Millipore, Billerica, MA, USA), primary antibodies, respectively.

### Quantification of *NR4A1* transcriptional activity in primary mouse hepatocytes

Primary mouse hepatocytes were cultured for 24 h. The cells were treated with 20 μg/mL mouse ApoA-IV or an equivalent volume of PBS (vehicle control), after which the levels of NR4A1 mRNA and protein were measured at 0.5, 1, 2, 4, 6, and 8 h posttreatment. The amount of NR4A1 mRNA was measured by qRT-PCR using the NR4A1 forward (5'-CTGTCCGCTCTGGTCCTC-3') and NR4A1 reverse (5'-AATGCGATTCTGCAGCTCTT-3') primers.

### Analysis of the affects of ApoA-IV on NR4A1 expression in mice

Thirteen- to fourteen-week-old male C57BL/6J mice (Jackson Laboratory, Bar Harbor, ME, USA) were randomly divided into the fasted and fed groups (n = 5 per group). Food was withheld from the mice in the fasted group from 07:00 to 12:00, and food was provided to the fed group ad libitum during the same period. The mice in both groups were injected intraperitoneally with 1 μg/g of r-m-apoA-IV or saline (vehicle control) after the 5-h period. Two hours after the ApoA-IV treatment, each mouse was sacrificed by cervical dislocation, and the liver was harvested to assess the expression of NR1D1 or NR4A1 mRNA and protein using qRT-PCR and western blotting. This study was approved by the ethics committee of University of Cincinnati.

### Statistical analysis

Three or four replicates were used in each experiment, and the data are presented as the mean ± SE from at least three independent experiments. The intergroup differences were compared using unpaired two-tailed Student *t-*tests. The level of statistical significance was set a *P* < 0.05. The data for figures used in these analyses is available in S1 Table.

## Results

### Colocalization of ApoA-IV with NR4A1 in cultured cells

**Fig 1:**
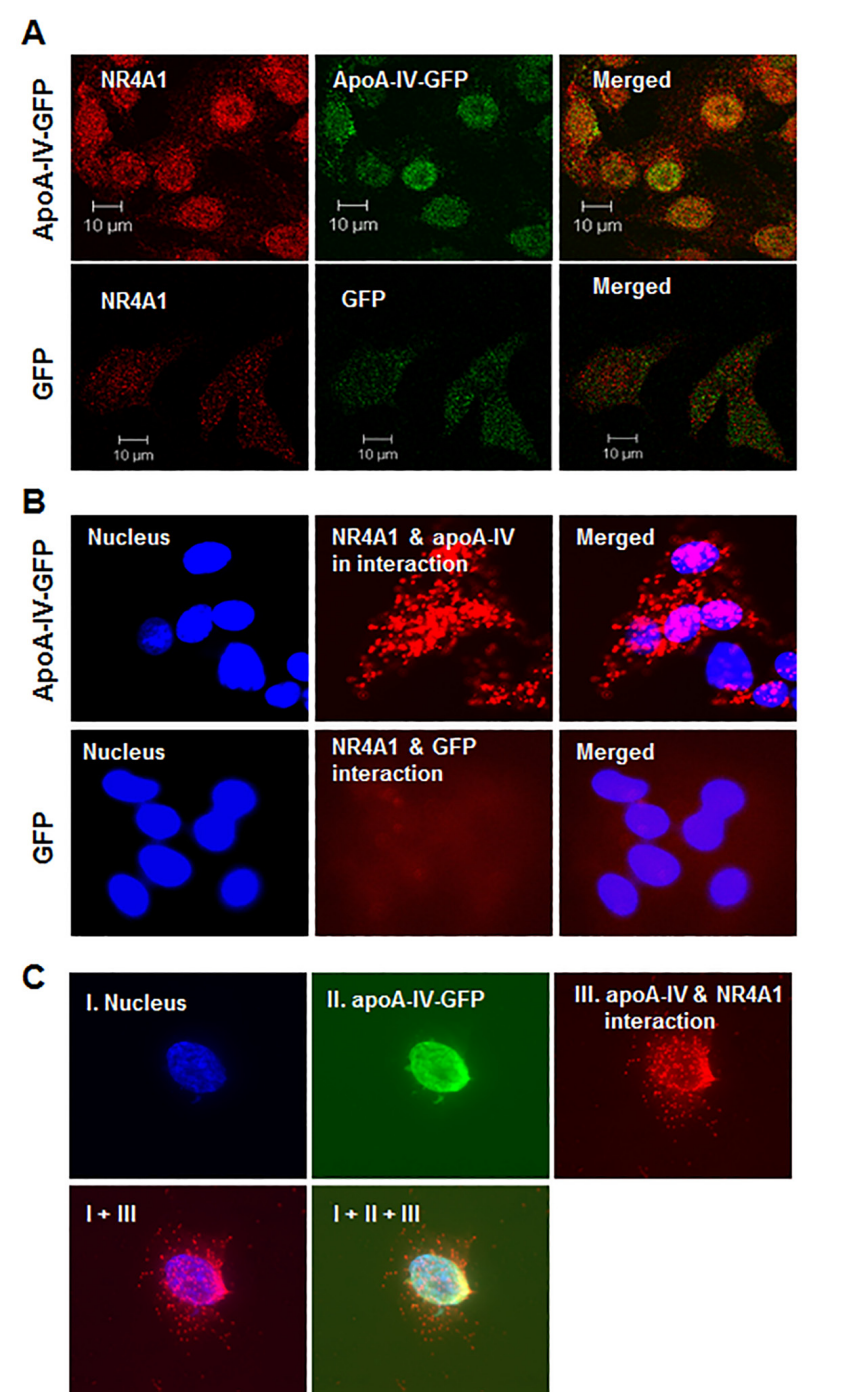
Colocalization of apoA-IV and NR4A1. **(A)** Subcellular colocalization of apoA-IV and NR4A1 was detected by immunofluorescence. HepG2 cells were transfected with the human NR4A1 expression plasmid for 48 h, and treated with r-h-apoA-IV-GFP (20 μg/mL) or GFP protein (negative control) for 2 h. The cells were probed with rabbit anti-human NR4A1 and mouse anti-GFP primary antibodies, followed by Alex Flour-594-conjugated goat anti-rabbit (red) and FITC-conjugated goat anti-mouse secondary antibodies, respectively. The cells were examined using a Zeiss LSM-510 confocal fluorescence microscope. **(B and C)** Colocalization of apoA-IV and NR4A1 was confirmed by PLA (Materials and Methods). **(B)** HepG2 cells and **(C)** HEK-293 cells were transfected as described above for immunofluorescence. The PLA was performed using anti-NR4A1 and anti-GFP primary antibodies, generating fluorescent red foci when examined using a Zeiss Axiovert 200 fluorescence microscope, which indicated the presence of both NR4A1 and r-h-apoA-IV-GFP in close proximity. The cells were counterstained with DAPI (blue) to visualize the nucleus.

**Fig 2:**
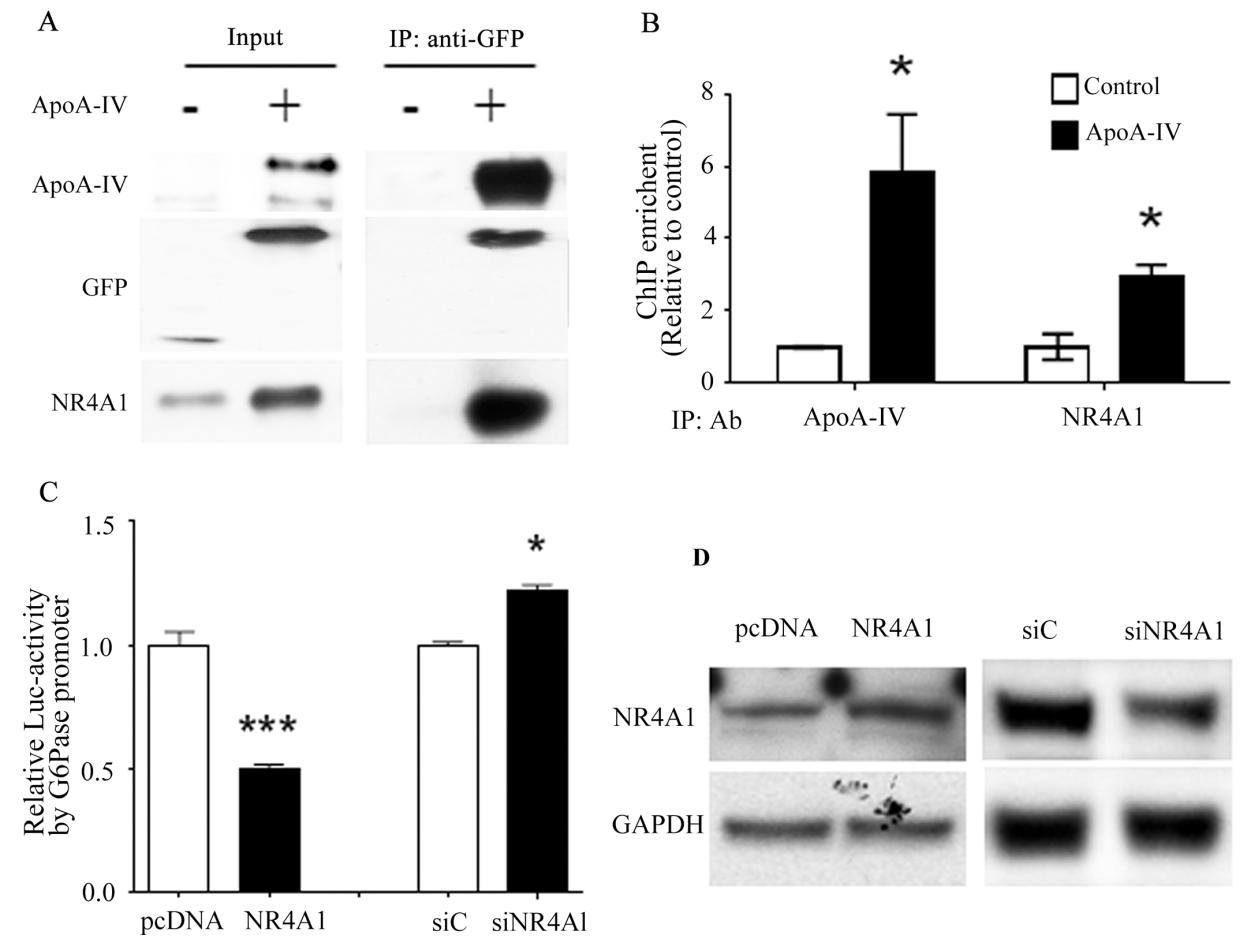
Effect of NR4A1 on the ApoA-IV-mediated regulation of *G6Pase* promoter activity. **(A)** HepG2 cells were treated with r-h-apoA-IV-GFP or GFP (control) for 6 h. Nuclear proteins were extracted from the cells, and immunoprecipitation was performed using an anti-GFP antibody. The precipitates were analyzed for the presence of ApoA-IV-GFP and NR4A1 by western blotting. **(B)** The colocalization of exogenous ApoA-IV and endogenous NR4A1 at the human *G6Pase* promoter was detected using ChIP. HepG2 cells were treated as described above for coimmunoprecipitation. Immunoprecipitation was performed using anti-NR4A1 and anti-apoA-IV antibodies. Primers were used to amplify the RORE sequence in the human *G6Pase* promoter or the *GAPDH* promoter (control) by PCR. The mean ± SE of three samples is shown (*P < 0.05 vs. vehicle control). **(C)** The effect of NR4A1 on *G6Pase* transcription in HEK-293 cells was examined using a luciferase reporter assay. HEK-293 cells were transfected with the *G6Pase*-luciferase reporter plasmid, human NR4A1 plasmid, renilla luciferase control reporter plasmid, and siNR4A1, control siRNA, or an equivalent volume of solvent for 24 h. Cells were also transfected with the G6Pase-luciferase and renilla luciferase reporter plasmids and the pcDNA3.1 plasmid as a control. The transfected cells were treated with recombinant human ApoA-IV protein or vehicle control for 24 h, and relative luciferase activity was measured. Relative luciferase activities (right) are presented as the mean ± SE of at least three samples from three independent experiments (***P < 0.001 vs. pcDNA or siC controls). **(D)** Western blotting of cells transfected with the pcDNA plasmid and no siRNA (pcDNA), cells transfected with the NR4A1 expression plasmid and no siRNA (NR4A1), cells cotransfected with the control siRNA (siC), and cells cotransfected with the siNR4A1 (siNR4A1).

To test whether NR4A1 is involved in the ApoA-IV-mediated regulation of hepatic glucose metabolism, we examined whether ApoA-IV colocalized with NR4A1. The immunofluorescence experiments showed that HepG2 and HEK-293 cells transfected with the human *NR4A1* plasmid and subsequently treated with r-h-apoA-IV-GFP exhibited a high level human NR4A1 expression and colocalization of ApoA-IV and NR4A1, primarily in the nucleus (Fig 1A–1C). The PLA experiments also showed that ApoA-IV colocalized with NR4A1 primarily in the nucleus of HepG2 cells, with a lower level of cytoplasmic colocalization (Fig 1B). The location of high-density ApoA-IV staining exactly overlapped that of NR4A1 in HEK-293 cells (Fig 1C). These data suggested that ApoA-IV was taken up by cells, and that ApoA-IV colocalized with NR4A1in the nucleus and to a lesser extent in the cytoplasm. To further investigate the interaction of ApoA-IV with NR4A1 in the nucleus of hepatocytes, we immunoprecipitated nuclear proteins from HepG2 cells treated with r-h-apoA-IV-GFP using anti-GFP antibodies, and both ApoA-IV and NR4A1 were identified in the precipitates (Fig 2A). These results further confirmed that ApoA-IV colocalizes with NR4A1 in the nucleus of hepatocytes.

### NR4A1 suppresses *G6Pase* promoter activity

Our previous study showed that ApoA-IV represses the expression of the gluconeogenic enzymes, G6Pase and PEPCK, in hepatocytes, which lowers hepatic glucose production both *in vitro* and *in vivo* [23]. In our current study, the chromatin immunoprecipitation (ChIP) experiments showed that ApoA-IV and NR4A1 colocalize near the RORE sequence in the *G6Pase* promoter (Fig 2B). This result implies that ApoA-IV and NR4A1 colocalize at the promoters of gluconeogenic genes in hepatocytes. To determine whether NR4A1 influences ApoA-IV-mediated repression of gluconeogenic genes at the transcriptional level, we examined the effect of NR4A1 on *G6Pase* gene expression in HEK-293 cells using a luciferase reporter assay. *G6Pase* promoter activity was significantly lower in the HEK-293 cells transfected with the NR4A1 expression plasmid and no siRNA, compared to those transfected with pcDNA3.1 plasmid, whereas luciferase expression was significantly higher in the cells in which NR4A1 expression was knocked down by cotransfection with siNR4A1, compared with those transfected with the control siRNA (Fig 2C). Western blotting confirmed that the level of NR4A1 was higher in the cells transfected with the NR4A1 expression plasmid and no siRNA, compared to that in the cells transfected with the pcDNA plasmid and no siRNA, and the level of NR4A1 was knocked down approximately 3-fold by the siNR4A1 treatment in the cells cotransfected with the siNR4A1, compared with that in the cells cotransfected the control siRNA (Fig 2D). These data suggest that the overexpression of NR4A1 mediated the decrease in G6Pase transcription, whereas reduced NR4A1 expression increased G6Pase transcription.

### NR4A1 suppresses G6Pase and PEPCK mRNA expression

**Fig 3:**
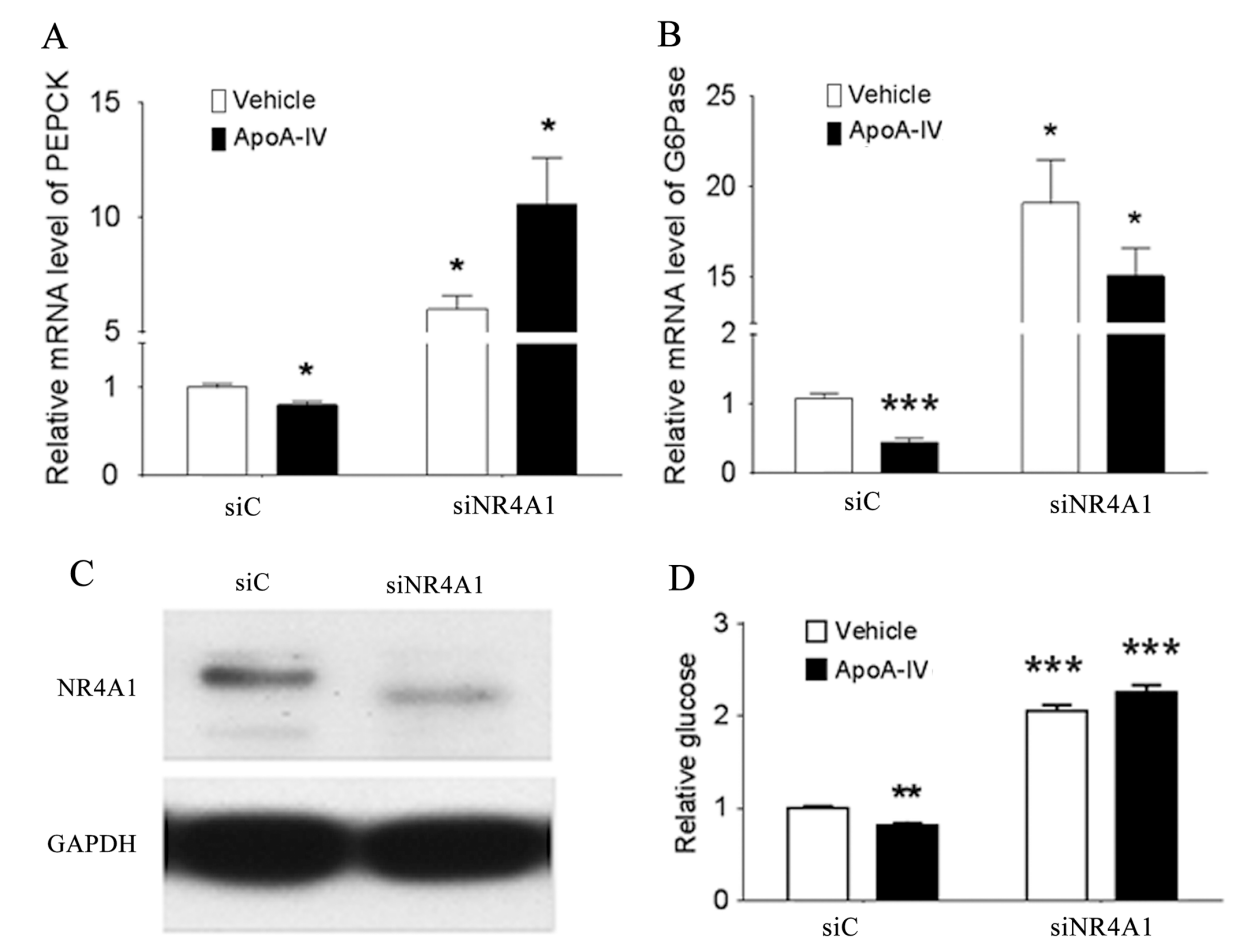
Effect of NR4A1 on ApoA-IV-mediated regulation of G6Pase and PEPCK expression and glucose production. Primary mouse hepatocytes were transfected with siNR4A1 or control siRNA (siC) for 48 h, and treated with 20 μg/mL r-m-apoA-IV or an equivalent volume of PBS (vehicle control) for 6 h or overnight. The levels of the **(A)** PEPCK and **(B)** G6Pase mRNAs were measured by qRT-PCR at 6 h posttreatment. **(C)** The levels of the NR4A1 and GAPDH (control) proteins were assessed by western blotting to demonstrate the inhibition of NR4A1 protein expression at 6 h posttreatment. **(D)** The level of glucose in the culture medium was also measured at 24 h posttreatment. Data are presented as the mean ± SE of at least three samples from three independent experiments (*P < 0.05, **P < 0.01, and ***P < 0.001 vs. vehicle or siC control).

Because the colocalization of ApoA-IV and NR4A1 reduced the transcriptional activity of the *G6Pase* promoter *in vitro*, we investigated whether the loss of *NR4A1* gene function affected the ApoA-IV-mediated expression of G6Pase and PEPCK and glucose production in primary mouse hepatocytes. The levels of the PEPCK and G6Pase mRNAs (Fig 3A and 3B) and glucose output (Fig 3C) were lower in the cells treated with r-m-apoA-IV (ApoA-IV/siC), compared with the control cells (vehicle/siC). However, the levels of the PEPCK and G6Pase mRNAs were 6.71- and 19.08-fold higher (*P* < 0.05), respectively, in the r-m-apoA-IV-treated cells in which the expression of NR4A1 was knocked down, compared to those of the ApoA-IV-treated control cells (Fig 3A and 3B). Glucose output was 2.02-fold higher in the ApoA-IV-treated cells in which the expression of NR4A1 was knocked down via RNAi, compared with that in the ApoA-IV-treated control cells (Fig 3D). These data indicate that NR4A1 downregulates *G6Pase* and *PEPCK* gene expression. These results collectively suggest that the colocalization of ApoA-IV and NR4A1 at the *PEPCK* and *G6Pase* promoters represses gluconeogenic gene expression in hepatocytes and reduces glucose output.

### Regulation of NR4A1 and NR1D1 expression

**Fig 4:**
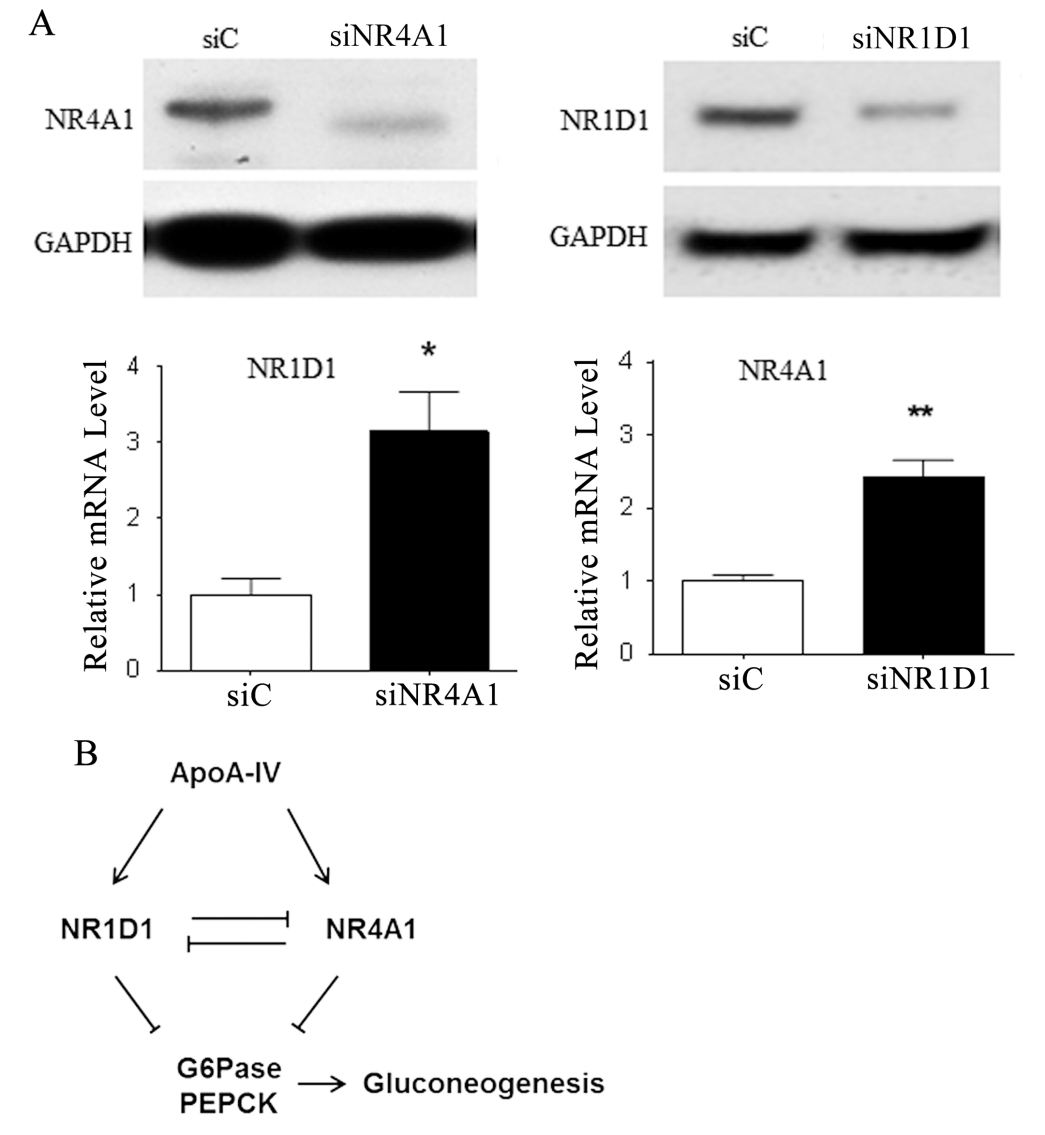
Mutual regulation of NR4A1 and NR1D1 expression in mouse hepatocytes. **(A)** Primary mouse hepatocytes were transiently transfected with siNR4A1, siNR1D1, or control siRNA (siC) for 48 h. **(Lower panel)** The levels of the NR1D1 and NR4A1 mRNAs were measured, relative to the cyclophilin control, using qRT-PCR. **(Upper panel)** The levels of the NR1D1 and NR4A1 proteins were measured, relative to the GAPDH control, by western blotting. Data are presented as the mean ± SE of at least three samples from three independent experiments (*P < 0.05, and **P < 0.01 vs. vehicle or siC control). **(B)** Diagram depicting the roles of ApoA-IV, NR4A1, and NR1D1 in the downregulation of hepatic glucose production. The ApoA-IV-induced expression of NR4A1 and NR1D1 represses the transcription of G6Pase and PEPCK in hepatocytes, which in turn reduces glucose output. The expression of NR4A1 and NR1D1 is also regulated by bidirectional feedback, in which each NR represses the expression of the other.

To further clarify the relationship between NR4A1 and NR1D1 in hepatic glucose metabolism, we examined how the level of NR4A1 expression affected that of NR1D1 and *vice versa* using RNAi and qRT-PCR. We found that siRNA-mediated knock down of NR4A1 expression increased the level of the NR1D1 mRNA in primary mouse hepatocytes, relative to that in the cells treated with the control siRNA, and that knocking down NR1D1 expression increased the relative level of the NR4A1 mRNA (Fig 4A). Western blot analysis of the cells treated with and without siRNA confirmed the qRT-PCR results (Fig 4A). These data suggest that NR1D1 and NR4A1 each downregulate the expression of the other, and that they may be competitive binders of ApoA-IV that serve similar or complementary functions (Fig 4B).

### ApoA-IV induces expression of NR4A1 *in vitro* and *in vivo*

**Fig 5:**
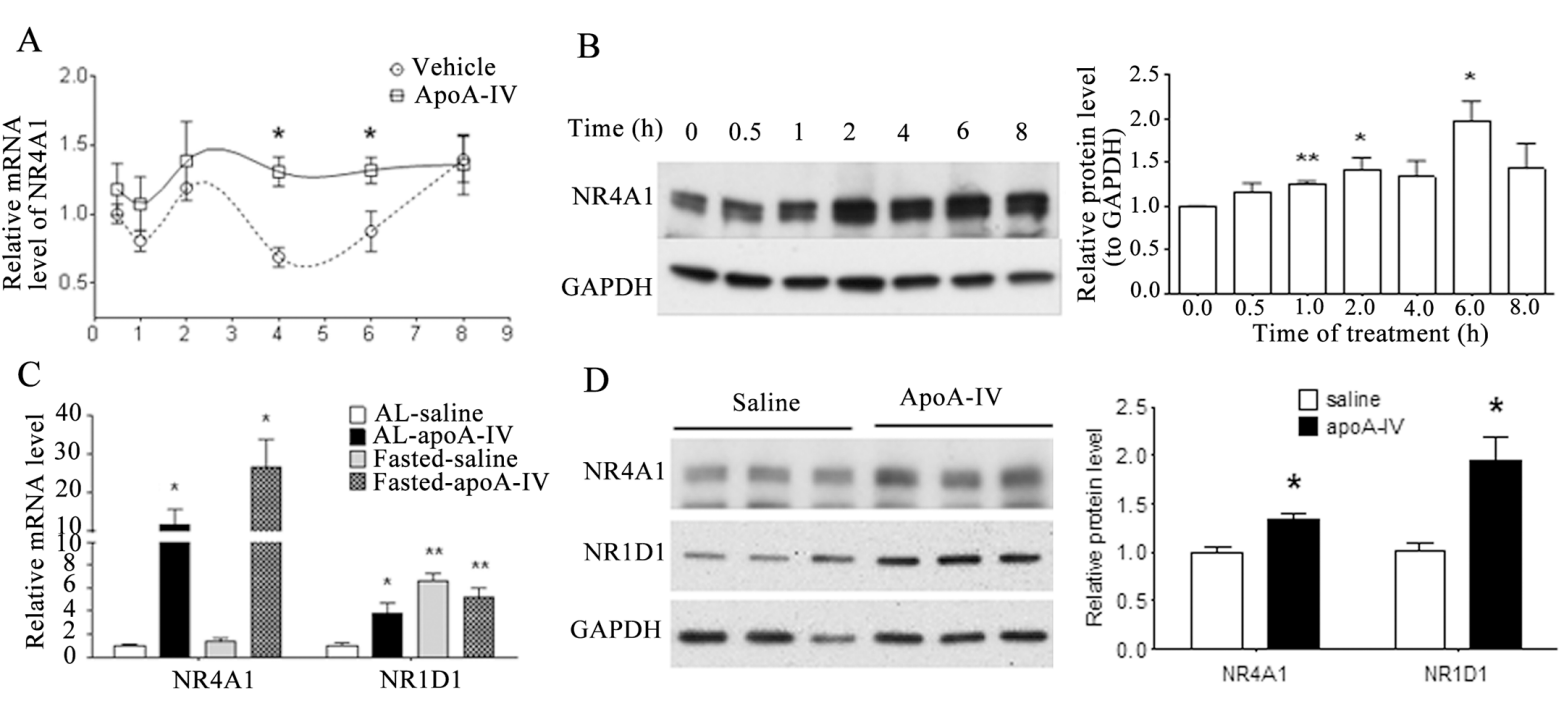
Effect of ApoA-IV treatment on NR4A1 gene expression in cultured cells *in vitro* and *in vivo*. Primary mouse hepatocytes were treated with 20 μg/mL r-m-ApoA-IV or an equivalent volume of PBS (vehicle control). The level of NR4A1 **(A)** mRNA and **(B)** protein expression were measured relative to the cyclophilin control using qRT-PCR and western blotting, respectively. The data from three independent experiments are presented as the mean ± SE of at least three samples from three independent experiments (*P < 0.05 and **P < 0.01 vs vehicle). Mice (n = 5 each group) were provided food ad libitum (AL) or fasted for 5 h before receiving an intraperitoneal injection of 1 μg/g r-m-ApoA-IV protein or saline (vehicle control). At 2 h postinjection, the levels of NR1D1 and NR4A1 **(C)** mRNA and **(D)** protein expression were measured, relative to the cyclophilin or GAPDH control, a using qRT-PCR and western blotting, respectively (*P < 0.05 and **P < 0.01 vs saline controls).

To further investigate how NR4A1 expression influences the effect of ApoA-IV on glucose metabolism, we examined the levels of NR4A1 mRNA and protein in primary mouse hepatocytes treated with r-m-apoA-IV. We found that the level of NR4A1 mRNA was significantly higher at both 4 h (1.9-fold) and 6 h (1.5-fold) after treatment with exogenous r-m-apoA-IV (Fig 5A), compared with those in the vehicle control cells. The level of NR4A1 protein increased at 1 h after treatment with r-m-apoA-IV, and reached a maximum level at 6 h after r-m-apoA-IV treatment (Fig 5B). Because the expression of NR1D1 and the binding of NR1D1 by ApoA-IV regulates hepatic gluconeogenic gene expression [23], we investigated whether the ApoA-IV-mediated downregulation of hepatic gluconeogenesis in mice influences the expression NR4A1 and NR1D1. The level of NR1D1 was significantly higher in the livers of mice in the fasted group, compared with that in the fed group, whereas the level of NR4A1 mRNA was not significantly different between the fed and fasted groups (Fig 5C). Following treatment with r-m-apoA-IV, the levels of the NR4A1 mRNA (Fig 5C) and protein (Fig 5D) significantly increased in both the fed and fasted groups, relative to the vehicle control mice. By contrast, the level of NR1D1 mRNA increased in the fed group following the ApoA-IV treatment, relative the vehicle control, whereas the difference in the level of NR1D1 mRNA in the ApoA-IV- and saline-treated mice was not significant in the fasted group. These data indicate that ApoA-IV upregulates NR4A1 expression in the downregulation of gluconeogenesis.

## Discussion

ApoA-IV is secreted primarily by the intestine in response to eating, and circulates in plasma as a complex containing cholesterol and phospholipids [24]. Multiple types of cells and tissues are affected by ApoA-IV, including kidney cells, skin fibroblasts, aortic endothelia cells, macrophages, preadipocytes, and hepatocytes [25–28]. The mechanism by which cells import ApoA-IV from plasma and the downstream effectors of ApoA-IV signaling in target cells have long remained unclear. However, our recent previous study showed that ApoA-IV colocalizes with NR1D1, which downregulates gluconeogenesis [23]. In our current study, we found that ApoA-IV also colocalizes with NR4A1, which stimulates the expression of NR4A1 and represses hepatic glucose production.

The transient expression of NR4A1 is rapidly induced in a wide range of tissues and cultured cells by various stimuli, including various pathways mediated by receptor agonists. The ligand-binding domain of NR4A1 does not form the cavity structure that is characteristic of ligand-activated NRs, suggesting that orphan NR function is ligand independent [29], and no endogenous ligands have been shown to interact with NR4A1 in gluconeogenesis. However, NR4A1 transcriptional activity in mammalian cells correlates with a more stable conformation of the NR4A1 ligand-binding domain [29].

In our current study, the immunofluorescence data showed that treating cells with exogenous ApoA-IV induced the ectopic expression of NR4A1 protein in human liver (HepG2) cells and human kidney (HEK-293) cells, and that ApoA-IV and NR4A1 colocalized primarily in the nucleus. The immunofluorescence data clearly showed that ApoA-IV is imported into the cytoplasm and nucleus of human hepatocytes, and that ApoA-IV colocalizes with NR4A1 in the nucleus and, to a lesser extent, in the cytoplasm of HepG2 and HEK-293 cells. These data also suggested that ApoA-IV upregulates the expression of NR4A1. Our coimmunoprecipitation, ChIP, and PLA data also showed that ApoA-IV colocalized with NR4A1 in the nucleus, which suggests that ApoA-IV might function as an NR4A1 ligand in hepatocytes. The pattern of ApoA-IV and NR4A1 colocalization differed from that of ApoA-IV and NR1D1 [23], suggesting that the mechanisms of colocalization and nuclear translocation differ between NR1D1 and NR4A1.

The results of our luciferase reporter experiments showed that ApoA-IV and NR4A1 repressed the transcriptional activity of the *G6Pase* promoter, and that the siRNA-mediated knock down of NR4A1 expression increased *G6Pase* transcriptional activity. Combined with the ChIP data, these results suggest that ApoA-IV downregulates G6Pase expression through the colocalization of ApoA-IV and NR4A1 at the *G6Pase* promoter. The *G6Pase* promoter contains an NGFI-B response element (NBRE) sequence (AAAGGTCA) and a ROR response element sequence (CTGACCTTGATTT) that are bound by the NR4A1 and NR1D1 proteins, respectively [22,30]. However, whether NR1D1 and NR4A1 competitively bind to the *G6Pase* promoter or function in a complementary manner to regulate G6Pase expression is unclear.

In a previous study, Pei et al. showed that the induction of adenoviral NR4A1 expression via the activation of the cAMP axis in response to glucagon and prolonged fasting induced the expression of gluconeogenic genes, stimulated hepatic glucose production, and raised blood glucose levels *in vivo* [30]. We performed gain- and loss-of-function experiments in mouse primary hepatocytes to investigate whether NR4A1 influenced the effect of ApoA-IV on hepatic glucose metabolism. In contrast to the findings of Pei et al. [30], our results showed that the overexpression of NR4A1 repressed the transcriptional activity of the *G6Pase* promoter, and that the levels of G6Pase and PEPCK expression were elevated by knocking down NR4A1 expression. These findings are consistent with our previous report of the repression of hepatic gluconeogenesis by ApoA-IV and NR1D1 [23].

In our current study, we also found that ApoA-IV-induced NR4A1 expression in primary mouse hepatocytes. Therefore, we investigated whether ApoA-IV-induced NR1D1 and NR4A1 expression during the repression of gluconeogenic genes *in vivo*. Our animal experiments showed that intraperitoneally administered ApoA-IV induced NR4A1 expression both in fed and fasted mice, whereas it induced NR1D1 expression in fed mice only. These data indicate that ApoA-IV is a positive regulator of NR4A1 expression, and that NR1D1 and NR4A1 influence different regulatory mechanisms. These findings can be partially explained by our observation that each of these NRs inhibits the expression of the other. These data suggest that the ApoA-IV-induced expression of NR4A1 and NR1D1 represses the transcription of certain gluconeogenic genes, and that the effects of NR4A1 and NR1D1 are complementary and feedback regulated, as depicted in the diagram in Fig 4B.

Transcriptional control of gluconeogenic gene expression involves multiple hormonal signals, second messenger pathways, and downstream effectors [31]. The diversity of the pathways involved in the regulation of NR1D1 and NR4A expression suggest that the biological roles of these NRs are cell-specific and highly dependent on the physiological context [32–35]. The findings of our current study provide important new information about the link between ApoA-IV and NR4A1 and their roles in hepatic glucose metabolism. During feeding, the serum level of insulin rises as ApoA-IV expression increases, and the level of glucagon drops. Our results show that the ApoA-IV-NR1D1/NR4A1 pathway counteracts gluconeogenic stimuli, and downregulates hepatic gluconeogenesis. Future studies of the effects of ApoA-IV on glucose metabolism in hepatocytes are warranted to further clarify the NR4A- and NR1D1-mediated regulatory mechanisms of gluconeogenic gene expression. Future studies are also warranted to investigate possible roles for the ApoA-IV-NR1D1/NR4A1 pathway in lipid metabolism.

In conclusion, we had previously shown that ApoA-IV expression suppressed gluconeogenic gene expression and reduced serum glucose in both in fed and fasted mice without increasing insulin secretion [23]. In our current study, we found that the orphan NRs, NR1D1 and NR4A1, mediated the effects of ApoA-IV on hepatic glucose production in cultured human and mouse hepatocytes and in mice. Our results showed that ApoA-IV was internalized by hepatocytes, and that intracellular ApoA-IV colocalized with both NR1D1 and NR4A1, and that the colocalization of ApoA-IV with NR1D1 and NR4A1 suppressed the transcription of the key gluconeogenic genes, *G6Pase* and *PEPCK*. ApoA-IV also increased the expression of NR4A1, which further suppressed gluconeogenesis. These ApoA-IV-mediated events decreased hepatic glucose production, which lowered blood glucose.

## Supporting Information

S1 ARRIVE Checklist ARRIVE Guidelines Checklist. Animal Research: Reporting In Vivo Experiments.
(PDF)

S1 Table Data for Figures. (DOCX)
