# Supplementary material for: Interaction of ApoA-IV with NR4A1 and NR1D1 Represses G6Pase and PEPCK Transcription: Nuclear Receptor-Mediated Downregulation of Hepatic Gluconeogenesis in Mice and a Human Hepatocyte Cell Line
Source: PLoS One. 2015 Nov 10;10(11):e0142098. doi: 10.1371/journal.pone.0142098 (PMC4640595; doi:10.1371/journal.pone.0142098)
Supplement: S1 Table — (DOCX) [file pone.0142098.s002.docx]

**1. Data for Fig2B**

|  | Control | | | | ApoA-IV | | | | test |
| --- | --- | --- | --- | --- | --- | --- | --- | --- | --- |
| ApoA-IV | 0.9756 | 1.0457 | 0.9209 | 1.045 | 7.8051 | 8.96572 | 4.80461 | 1.7184 | 0.037507 |
| NR4A1 | 1.3104 | 0.285 | 1.404 |  | 3.4581 | 2.44526 | 3.01 |  | 0.0142 |

**2. Data for Fig2C**

|  | pcDNA | Nr4a1 | siC | siNr4a1 |
| --- | --- | --- | --- | --- |
|  | 0.83 | 0.52 | 1.03 | 1.14 |
|  | 1.12 | 0.56 | 1.02 | 1.18 |
|  | 1.18 | 0.5 | 1.01 | 1.19 |
|  | 0.87 | 0.5 | 1.01 | 1.28 |
|  | 1.03 | 0.48 | 0.95 | 1.21 |
|  | 0.96 | 0.44 | 0.98 | 1.31 |
| Test |  | 0.000168 |  | 0.000128 |

**3. Data for Fig3A**

| PEPCK | Vehicle | | | ApoA-IV | | | test |  |
| --- | --- | --- | --- | --- | --- | --- | --- | --- |
| scr | 0.99 | 1.08 | 0.93 | 0.87 | 0.81 | 0.72 | 0.031541 | 0.013697 |
| siNr4a1 | 6.99 | 4.92 | 6.09 | 6.51 | 12.2 | 13 | 0.146109 | 0.042625 |

**4. Data for Fig3B**

| G6Pase | Vehicle | | | | ApoA-IV | | | | | | Test |  |
| --- | --- | --- | --- | --- | --- | --- | --- | --- | --- | --- | --- | --- |
| siC | 0.98 | 0.98 | 1.05 | 1.29 | 0.42 | 0.49 | 0.64 | 0.24 | 0.56 | 0.3 | 0.000362 | 0.017132 |
| siNr4a1 | 17.94 | 15.62 | 23.67 |  | 14.57 | 17.94 | 12.69 |  |  |  | 0.242773 | 0.011691 |

**5. Data for Fig3D**

| Glucose | Vehicle | | | | ApoA-IV | | | | test |  |
| --- | --- | --- | --- | --- | --- | --- | --- | --- | --- | --- |
| siC | 0.97 | 0.94 | 1.04 | 1.04 | 0.71 | 0.84 | 0.84 | 0.84 | 0.004225 | 0.000151 |
| siNr4a1 | 2.17 | 2.17 | 1.94 | 1.94 | 2.08 | 2.31 | 2.16 | 2.46 | 0.117268 | 0.000294 |

**6. Data for Fig4**

|  | NR1D1 | | NR4A1 | |
| --- | --- | --- | --- | --- |
|  | siC | siNr4a1 | siC | siNr1d1 |
|  | 0.115 | 3.199 | 1.207 | 2.587 |
|  | 1.393 | 1.838 | 0.743 | 2.101 |
|  | 1.493 | 2.111 | 1.05 | 1.707 |
|  | 1.227 | 4.579 | 1.243 | 2.664 |
|  | 1.068 | 3.987 | 0.879 | 3.06 |
|  | 0.705 |  | 0.879 |  |
| Test |  | 0.011707 |  | 0.002346 |

**7. Data for Fig5A**

| Time | Vehicle | | | | | | ApoA-IV | | | | | | test |
| --- | --- | --- | --- | --- | --- | --- | --- | --- | --- | --- | --- | --- | --- |
| 0.5 | 1.07 | 0.93 | 1 |  |  |  | 1.42 | 0.81 | 1.31 |  |  |  | 0.440199 |
| 1 | 0.73 | 0.73 | 0.96 |  |  |  | 1.27 | 0.68 | 1.27 |  |  |  | 0.308084 |
| 2 |  | 1.19 | 1.19 |  |  |  | 1.93 | 1.27 | 0.96 |  |  |  | 0.562747 |
| 4 | 0.78 | 0.55 | 0.73 |  |  |  | 1.1 | 1.46 | 1.36 |  |  |  | 0.012184 |
| 6 | 0.68 | 1.36 | 0.59 | 0.7 | 1.31 | 0.61 | 0.99 | 1.51 | 1.31 | 1.31 | 1.16 | 1.63 | 0.032827 |
| 8 |  |  |  | 1.73 | 1.23 | 1.23 |  |  |  | 1.63 | 0.93 | 1.52 | 0.900377 |

**8. Data for Fig5B**

| Time | 0.0 | 0.5 | 1.0 | 2.0 | 4.0 | 6.0 | 8.0 |
| --- | --- | --- | --- | --- | --- | --- | --- |
|  | 1.004077 | 1.08 | 1.25 | 1.27 | 1.33 | 1.56 | 1.56 |
|  | 0.998525 | 1.21 | 1.35 | 1.72 | 1.85 | 2.34 | 2.19 |
|  | 1 | 0.96 | 1.17 | 1.35 | 1.07 | 2.02 | 1.02 |
|  | 1 | 1.42 | 1.23 | 1.32 | 1.11 |  | 0.96 |
|  |  | 0.188642 | 0.006854 | 0.027583 | 0.154831 | 0.050122 | 0.228557 |

**9. Data for Fig5C**

|  | AL-saline | | | | | AL-apoA-IV | | | | | Fasted-saline | | | | | Fasted-apoA-IV | | | | |
| --- | --- | --- | --- | --- | --- | --- | --- | --- | --- | --- | --- | --- | --- | --- | --- | --- | --- | --- | --- | --- |
| Nr4a1 | 0.62 | 1.16 | 1.33 | 1.08 | 0.82 | 18.5 | 7.01 | 21.4 | 4.96 | 3.27 | 2.312286 | 0.94 | 0.29 | 1.42 | 1.75 | 26.2 | 26.2 | 1.33 | 45.5 | 34.5 |
| Nr1d1 | 1.67 | 0.68 | 0.12 | 1.35 | 1.18 | 3.11 | 1.79 | 7.15 | 3.58 | 3.58 | 6.225867 | 7.15 | 5.81 | 8.8 | 5.42 | 4.72 | 4.72 | 8.22 | 5.06 | 3.34 |
|  |  |  |  |  |  |  |  |  |  | 0.054264 |  |  |  |  | 0.39627 |  |  |  |  | 0.024025 |
|  |  |  |  |  |  |  |  |  |  | 0.030276 |  |  |  |  | 0.000204 |  |  |  |  | 0.004578 |

**10. Data for Fig5D**

|  | Saline | | | ApoA-IV | | | | test |
| --- | --- | --- | --- | --- | --- | --- | --- | --- |
| NR4A1 | 0.890083 | 0.985585 | 1.092717 | 1.291086 | 1.151812 | 1.313324 | 1.357658 | 0.016509 |
| NR1D1 | 0.931473 | 0.799979 | 1.245039 | 1.188911 | 1.652942 | 1.605228 | 2.226042 | 0.045738 |
